# *Lactobacillus rhamnosus* Ameliorates Multi-Drug-Resistant *Bacillus cereus*-Induced Cell Damage through Inhibition of NLRP3 Inflammasomes and Apoptosis in Bovine Endometritis

**DOI:** 10.3390/microorganisms10010137

**Published:** 2022-01-10

**Authors:** Ning Liu, Xue Wang, Qiang Shan, Le Xu, Yanan Li, Bingxin Chu, Lan Yang, Jiufeng Wang, Yaohong Zhu

**Affiliations:** Department of Veterinary Clinical Sciences, College of Veterinary Medicine, China Agricultural University, Beijing 100193, China; nliu2224@163.com (N.L.); ivywang0913@163.com (X.W.); xiaoqiangdebaobao@163.com (Q.S.); xlyamiyou@163.com (L.X.); 13796685756@163.com (Y.L.); barrylao@163.com (B.C.); lan_ydth@126.com (L.Y.); jiufeng_wang@hotmail.com (J.W.)

**Keywords:** *Bacillus cereus*, probiotics, multidrug resistance, inflammasome, K^+^ efflux

## Abstract

*Bacillus cereus*, considered a worldwide human food-borne pathogen, has brought serious health risks to humans and animals and huge losses to animal husbandry. The plethora of diverse toxins and drug resistance are the focus for *B. cereus*. As an alternative treatment to antibiotics, probiotics can effectively alleviate the hazards of super bacteria, food safety, and antibiotic resistance. This study aimed to investigate the frequency and distribution of *B. cereus* in dairy cows and to evaluate the effects of *Lactobacillus rhamnosus* in a model of endometritis induced by multi-drug-resistant *B. cereus*. A strong poisonous strain with a variety of drug resistances was used to establish an endometrial epithelial cell infection model. *B. cereus* was shown to cause damage to the internal structure, impair the integrity of cells, and activate the inflammatory response, while *L. rhamnosus* could inhibit cell apoptosis and alleviate this damage. This study indicates that the *B. cereus*-induced activation of the NLRP3 signal pathway involves K^+^ efflux. We conclude that LGR-1 may relieve cell destruction by reducing K^+^ efflux to the extracellular caused by the perforation of the toxins secreted by *B. cereus* on the cell membrane surface.

## 1. Introduction

*Bacillus cereus* is a Gram-positive, endospore-forming, quiet soil dweller that thrives in a diversity of habitats or as part of the intestinal flora of different animals, or is widely distributed in natural environments and frequently found in foods, especially dairy products, and even persists in host epithelial cells [1,2,3,4,5,6,7], which can secrete several different bacterial toxins, including non-hemolytic enterotoxin (NHE), hemolysin BL (HBL), cytotoxin K (CytK), and metalloprotease immune inhibitor A (InhA). Moreover, *B. cereus* has a high isolation rate in dairy cows suffering from endometritis in China, which is often an aggravated infection involving isolation with pathogenic bacteria such as *Escherichia coli*, *Trueperella pyogenes*, *Fusobacterium necrophorum*, *Prevotella*, *Streptococcus*, and *Staphylococcus* species [8,9]. Most toxins of *B. cereus* belong to the family of pore-forming toxins (PFTs); among them, NHE and HBL are similar to the well-known cytolysin A (ClyA) of the α-PFT family, while CytK and hemolysins are members of the β-PFT family [10,11]. It is of particular concern for the food industry, as it causes food safety issues due to the formation of spores, biofilms, and diarrhea and/or emetic toxins [12]. As a common and ubiquitous food-borne pathogen, the presence of *B. cereus* and its virulence factors in dairy products may cause food poisoning and other serious diseases [13]. Previous studies have provided evidence that the HBL, NHE, and CytK cytotoxins are the main virulence factors in *B. cereus* related to the cause of diseases. However, their roles and process of damage are still unknown. Furthermore, *B. cereus* appears to contain many protease, peptide, and amino-acid transporter genes [11]. These observations indicate that *B. cereus* is better adapted to a protein diet, and animal tissues seem to provide a primary source of nutrients for it [14].

Antibiotic therapy is still the main method of clinical treatment of *B. cereus* infection. However, antibiotic abuse or acquisition of resistance genes through horizontal gene transfer are the main reasons for the emergence of antibiotic-resistant *B. cereus* strains [15,16], which can lead to a decrease in, or even the disappearance of, the therapeutic effect of some antibiotics [17,18]. In particular, the emergence of multidrug-resistant strains increases the chance of infection [19]. The consequences are unimaginable if antibiotic treatment is completely invalid. Hence, a detailed and effective investigation has to be conducted to raise the knowledge regarding antibiotic alternatives to treat *B. cereus* infection.

As a substitute for antibiotics, probiotics have a wide range of applications in treating animal diseases caused by bacterial infection. A large proportion of pathogenic bacteria secrete virulence genes mediating adhesion and colonization, by which the colonization of pathogenic bacteria on host cells or tissues is usually the initiation of infection. *Lactobacillus* can reduce pathogen adhesion to epithelial cells and exert direct antimicrobial activity due to the accumulation of antimicrobial substances [20]. The pretreatment of pregnant CD-1 mice with *L. rhamnosus* GR-1 culture supernatant decreases the lipopolysaccharide (LPS)-induced production of various cytokines and chemokines [21]. *Lactobacillus rhamnosus* GR-1, isolated from the healthy female urethra, is used to prevent urinary tract infections, preterm birth, and bacterial vaginosis [22]. Probiotics are live microorganisms that, when administrated in adequate amounts, confer a health benefit to the host [23]. The inhibitory effects of probiotics on Gram-negative bacteria have been studied a lot, but few studies have reported whether probiotics also have limited effects on Gram-positive bacteria, making research on Gram-positive bacteria even more essential.

Our objectives were to explore the mechanisms of *B. cereus* involved in causing damage to cells and to evaluate the ability of *L. rhamnosus* to alleviate cell damage induced by a strong virulent and multi-drug-resistant, clinically isolated *B. cereus*. In the present study, we show that *L. rhamnosus* GR-1 affects the disturbance of inflammatory proteins by regulating the NLR family pyrin domain containing 3 (NLRP3) pathway and preserves cell morphology and structure by increasing tight junction proteins and inhibiting apoptosis in bovine endometrial cells infected by *B. cereus*. We propose that *Lactobacillus rhamnosus* has an efficacious effect on alleviating the cell damage induced by the greatly virulent and multi-drug-resistant *B. cereus.* Our results indicate that *L. rhamnosus* GR-1 can be a potential alternative to antibiotics to treat uterine bacterial infection.

## 2. Materials and Methods

### 2.1. Biosecurity Statement

Clinical isolates of *Bacillus cereus* were treated strictly in accordance with the State Council of the People’s Republic of China regulations on the biological safety of pathogen microbiology laboratories (000014349/2004-00195). All necessary, safe operations were conducted to avoid pathogen transmission and infection.

### 2.2. Clinical Isolates of B. cereus Culture and Sequencing

All 39 clinical *B. cereus* strains were collected and isolated from the uterine lavage fluid of dairy cows between postpartum days 21 to 28 from three different dairy farms in northern China. The animals were aged between two and five years old, and the average parity was 2.5. There was no application of antibiotic treatment before the sample collection for any dairy cow. The uterine lavage fluid was evenly vortexed and diluted 10 times with normal saline. The original lavage fluid solution and the dilution were evenly spread on 5% sheep blood plates, which were incubated upside down in a 37 °C incubator for 12 h. Each concentration was duplicated. Preliminary screening was carried out according to the colony morphology characteristics and the presence of hemolysis loops. A single colony was cultivated on a new blood plate using an inoculating loop with the three-dimensional line method. After incubating at 37 °C for 12 h, a purified single colony was incubated in Luria–Bertani (LB) broth (Aobox, Beijing, China) for 12 h until the bacterial liquid became turbid.

The overnight cultured bacterial liquid DNA was extracted with a TIANamp bacterial DNA kit (DP302, TIANGEN, Beijing, China), and PCR (Applied biosystems, 9902, Waltham, MA, USA) amplification was performed using 16S gene primers (F: AACTGGAGGAAGGTGGGGAT, R: AGGAGGTGATCCAACCGCA). The PCR products were sent to Beijing Tianyi Huiyuan Biotechnology Co., Ltd. for sequencing. The sequencing results were compared and analyzed using the BLAST program (National Center for Biotechnology Information, Bethesda, MD, USA), and BC1908 was selected for the following experiments.

### 2.3. Determination of MIC

The Minimum Inhibitory Concentration (MIC) of *B. cereus* (BC1908) was determined by the broth microdilution method, and *Staphylococcus aureus* ATCC 29213 was used as the quality control reference. The test was operated strictly according to performance standards for antimicrobial susceptibility testing M100-S28 of the clinical and laboratory standards institute (CLSI). The MIC results for ampicillin, amoxicillin, azithromycin, cefazolin, enrofloxacin, gentamicin, kanamycin, streptomycin, and tetracycline were taken from three independent repetitions per trial. The isolates were defined as “susceptible,” “intermediate,” or “resistant” based on the MIC for each antimicrobial agent.

### 2.4. BC1908 De Novo Sequencing

According to the above method, the DNA of BC1908 was extracted and sent to Beijing Saimo Lily Biotechnology Co., Ltd. for de novo sequencing to analyze the virulence genes and drug resistance genes.

### 2.5. Virulence Gene Testing

After the bacterial DNA was extracted according to the above method, the virulence gene primers (Table 1) were used for PCR virulence gene detection. PCR products were analyzed via 1% agarose gel electrophoresis for 30 min at 125 V in 1× Tris–acetate–EDTA buffer, stained with ethidium bromide, and photographed under UV transillumination.

### 2.6. Bovine Endometrial Cell Culture

For the bovine endometrial cell (BEEC) culture, primary BEECs were obtained from the uterine horn [22] and processed using the method previously described [24]. The BEECs were cultured with Dulbecco’s Modified Eagle Medium/Ham’s F-12 medium (1:1) and supplied with 10% heat-inactivated fetal horse serum, 1% penicillin, and streptomycin (Invitrogen, Carlsbad, CA, USA), respectively. After flowing evenly into cell flasks, the BEECs were cultured at 37 °C in an incubator with a 5% CO_2_ atmosphere.

### 2.7. Lactobacillus rhamnosus GR-1 Culture

ATCC55826 was purchased from the American Type Culture Collection (Manassas, VA, USA) and grown in De Man, Rogosa, and Sharpe (MRS) broth (Aobox, Beijing, China) for 24 h at 37 °C under microaerophilic conditions [21]. LGR-1 was inoculated and grown in fresh MRS broth for 8 h at 37 °C with a starting ratio of 1:100 after passing the mid-log phase.

### 2.8. Lactate Dehydrogenase Assay

To assay cell death under the different treatments examined, the lactate dehydrogenase (LDH) levels were measured using an LDH Cytotoxicity Assay Kit (Beyotime Biotechnology, Shanghai, China) according to the manufacturer’s instructions.

### 2.9. Western Blotting

Different treated BEECs (same as LDH) were lysed in 1 mL of lysis buffer, composed of 1 mL of RIPA buffer and 10 μL of phenylmethanesulfonyl fluoride (Solarbio, Beijing, China). The resulting lysates were centrifuged at 13,000× *g* for 10 min at 4 °C to pellet the insoluble material, and the supernatants were used for Western blot analysis. The following primary antibodies were used: rabbit polyclonal anti-NLRP3 (1:1000 dilution, 19771-1-AP), rabbit polyclonal anti-ASC (also known as apoptosis-associated speck-like protein containing a caspase activation and recruitment domain; 1:500 dilution, 10500-1-AP) (ProteinTech Group, Rosemont, IL, USA), rabbit polyclonal anti-Occludin (1:2000 dilution, ab216327) (Abcam, Boston, MA, USA), mouse monoclonal anti-ZO-1 (1:1000 dilution, 66452-1-Ig), rabbit polyclonal anti-BAX (1:4000 dilution, 50599-Ig), rabbit polyclonal anti-Bcl-2 (1:1000 dilution, 12789-1-AP), rabbit polyclonal anti-PARP (1:1000, 13371-1-AP), mouse monoclonal anti-caspase-3 (1:1000 dilution, 66470-2-Ig), rabbit polyclonal anti-caspase 8 (1:1000 dilution, 13423-1-AP), rabbit polyclonal anti-caspase 9 (1:1000 dilution, 10380-1-AP) (ProteinTech Group, Rosemont, IL, USA), rabbit polyclonal anti-FAS (a pro-apoptotic TNF receptor (TNFR) superfamily member) (1:500, WL03376), rabbit polyclonal anti-SMAC (the second mitochondria-derived activator of caspase) (1:500, WL0834), and rabbit polyclonal anti-TNF-R1 (1:500, WL01414) (Wanlaibio, Shenyang, China). The mouse anti-β-actin (1:5000 dilution, 66009-1-Ig) was applied for verifying the equal sample loading. Horseradish peroxidase-conjugated affinipure goat anti-mouse IgG (H+L) (1:5000 dilution, SA00001-1) or goat anti-rabbit IgG (H+L) (1:5000 dilution, SA00001-2) were used as secondary antibodies. Densitometric values of the Western blot images were obtained from three independent experiments using ImageJ software (National Institutes of Health, Bethesda, MD, USA). The results are presented as the ratio of the NLRP3, ASC, Zonula Occludens-1 (ZO-1), Occludin, BCL-2-associated X apoptosis regulator (BAX), the B cell lymphoma 2 (Bcl-2), cleaved caspase-3, cleaved caspase-8, cleaved caspase-9, FAS, SMAC, or the tumor necrosis factor receptor 1 (TNF-r1) band intensity to that of β-actin. For inhibition studies, an increasing concentration of KCl at 5, 25, 50, and 75 mM (P5405, Sigma, Sanit Louis, MO, USA) was added to BEECs 30 min before BC1908 stimulation. To activate the canonical NLRP3 inflammasome as a control, BEECs were primed using 500 ng mL^–1^ ultrapure LPS from *E. coli* (L4391, Sigma) for 4 h and stimulated with 5 mM ATP (A6419-1g, Sigma, Sanit Louis, MO, USA) for 45 min.

### 2.10. Immunofluorescence

The BEECs were plated on glass coverslips in a 24-well flat-bottomed culture plate, and the treated BEECs were washed with PBS three times, incubated with a mitochondrial localization probe (200 nM Mito-Tracker Red CMXRos, Beyotime Biotechnology, C1049B) according to the manufacturer’s instructions, and then fixed with 4% paraformaldehyde for 15 min on ice. The cells were rinsed three times with PBS and then incubated for 60 min in a blocking solution (1 × PBS/5% goat serum/0.3% Triton X-100) to reduce the nonspecific background. After being washed with PBS three times, the cells were incubated with goat anti-rabbit IgG (H+L) and FITC conjugate (TRANSGEN, Beijing, China) for 1 h at room temperature and DAPI (Solarbio, Beijing, China) for 10 min at 37 °C. The cells were then covered with a coverslip, and the edges were sealed to prevent drying. The cells were observed and photographed using a Nikon Eclipse Ti-U inverted fluorescence microscope equipped with a Nikon DS cooled camera head (Nikon, Hitachi, Tokyo, Japan).

### 2.11. Data Analysis

Statistical analysis was performed using Prism 7 (GraphPad Sofeware Inc., San Diego, CA, USA). Qualitative data are expressed as mean ± standard error of mean value (SEM; *n* = 3 or 6). One-way analysis of variance (ANOVA) was applied to analyze statistically significant differences at *p* < 0.05, followed by Tukey’s post-hoc test with multiple comparisons and a single pooled variance.

## 3. Results

### 3.1. Cell Cytotoxicity of B. cereus

Quantitative measurement of the LDH method was used to determine the cell death rate after 3 h of *B. cereus* challenge (Supplementary Figure S1A). BC1908 caused great damage to cells, with the highest cell death rate, which may have originated from strong multiple virulence. BC1908 was selected as the experimental strain for follow-up experiments based on the above results. The results also indicate that the toxicity of *B. cereus* isolated from the same dairy farm was quite different. This difference may have been due to the number and expression level of the virulence genes carried by *B. cereus* isolated from the same dairy farm. It may also be that the phylogeny of *B. cereus* from the same farm is also distinguished, which requires more research to explore the causes of the different virulence gene levels.

### 3.2. Protective Effect of LGR-1 on Cells Infected by B. cereus with Different MOIs

Using previous experimental investigations (Supplementary Figure S1B), we found that BC1908 had strong cytotoxicity, even in low MOI conditions. In order to explore the lowest addition for probiotics to protect cells from the lethality of *B. cereus*, the LDH toxicity test was conducted, and the results are shown in Supplementary Figure S1B. When cells (2 × 10^5^ cells/well) were protected by LGR-1 (10^8^ /CFU) in advance for 3 h and then exposed to BC1908 (10^6^/CFU), the cell death rate was lowest, with a significant statistical difference.

### 3.3. Antibiotic Sensitivity and Virulence Gene Tests of Bacillus cereus

Antimicrobial susceptibility testing (Table 2) was also performed to better explore the characteristics of *B. cereus*. BC1908 appeared multi-drug-resistant to the antibiotics involved in this study, except for streptomycin and enrofloxacin. This shows the danger of antibiotic abuse, and alternatives to antibiotics deserve more research.

*B. cereus* secretes multiple toxins, including HBLA, HBLB, HBLD, NHEA, NHEB, NHEC, *cytK, hlyIII* (Hemolysin III), *hlyA* (HemolysinA), and *inhA*, which were detected at the rates of 81%, 85%, 92%, 97%, 100%, 97%, 79%, 16%, 28%, and 41%, respectively, among all 39 clinical *B. cereus* strains (Supplementary Figure S2). BC1908 is equipped with HBLA, HBLB, HBLD, NHEA, NHEB, NHEC, *cytK, inhA, hlyIII* (Hemolysin III), and *hlyA* (HemolysinA). Among them, HBLs and NHEs are the two most important groups of virulence genes that play a vital role in destroying the host.

### 3.4. LGR-1 Ameliorates B. cereus-Induced Destruction of Endometrial Epithelial Cells

**Figure 1:**
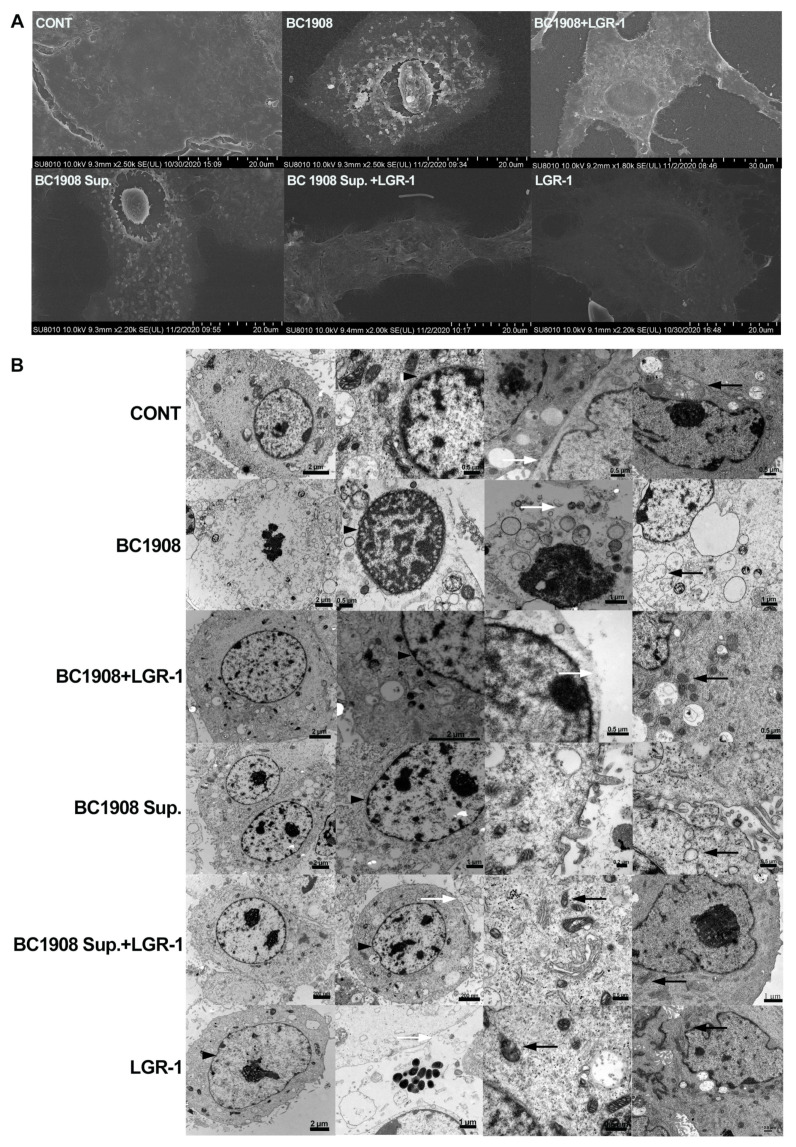
Scanning electron microscopy and transmission electron microscopy to observe LGR-1’s effects against *B. cereus* and BC1908 supernatant damage to the cell ultrastructure. (**A**) Scanning electron microscopy to observe the cell surface structure for different treatments. (**B**) Transmission electron microscopy to observe the changes in the cell ultrastructure in the different treatment groups. Black triangles indicate the nuclear membrane; white arrows indicate the cell membrane; black arrow refers to the mitochondria.

Scanning electron microscopy and transmitting electron microscopy were used to observe the mitigating effects of LGR-1 on the cell ultrastructure damage caused by *B. cereus* (Figure 1). In Figure 1A, it can be observed that cells challenged with the supernatants of BC1908 (BC1908 Sup.) and the BC1908 received serious damage, of which obvious inflammatory phenomena appeared on the cell surface, and the nucleus shrank and separated from the cytoplasm. Compared to the cells challenged by the BC1908 Sup. or BC1908 strain, the damage of cells protected by LGR-1 in advance was relieved significantly. LGR-1 had no obvious negative effects on the cells compared to the control cells.

The results of transmission electron microscopy (Figure 1B) show that the control cells and the cells exposed to LGR-1 alone retained their integrity. The mitochondria (indicated by black arrows), the cell nuclei (indicated by black triangles), and cell edges (indicated by white arrows) remained in a valuable condition. However, cells challenged with BC1908 and BC1908 Sup. were substantially disturbed. Both the nucleus and the cytoplasm showed dissolution. The border of the cell nucleus became blurred. Mitochondria were destroyed and leaked from the severely perforated cell membrane with other intracellular substances. Fortunately, the conditions of LGR-1-protected cells were enhanced compared to the conditions of BC1908- and BC1908 Sup.-challenged cells. Interestingly, the conditions of BC1908-infected cells show more severe damage in the nucleus and the mitochondria compared to the conditions of BC1908 Sup.-challenged cells.

### 3.5. Genetic-Level Analysis of B. cereus through Second-Generation Sequencing

**Figure 2:**
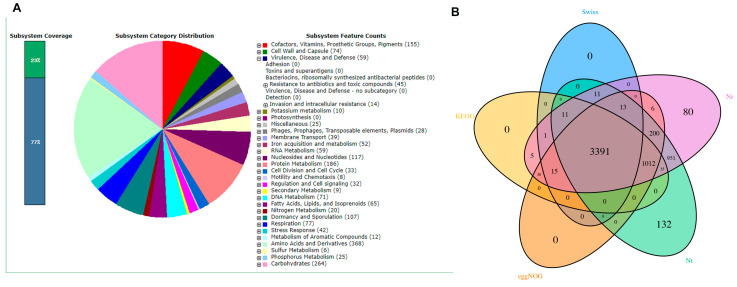
De novo sequencing results of the strong virulence of *B. cereus* (BC1908). (**A**) RAST analysis of BC1908 for virulence and resistance genes. (**B**) The intersection of analysis between different platforms (KEGG, eggNOG, Nt, Nr, and Swiss).

The sequencing results were analyzed on the RAST platform. *B. cereus* had several virulence and multidrug resistance genes. In Figure 2A, BC1908 gene sequencing was run on RAST (https://rast.nmpdr.org accessed on 22 December 2021). The results show that BC1908 is equipped with several virulence genes and resistance genes, which is consistent with the MIC and virulence gene detection results. In Figure 2B and Supplementary Figures S1 and S2, the results of the de novo sequencing of BC1908 run on different platforms to compare functions are shown. The BC1908 gene was enriched in the cellular process, binding, and catalytic activity, indicating that BC1908 is highly toxic to cells.

### 3.6. LGR-1 Alleviates the Inflammatory Response of BEECs Infected by B. cereus

**Figure 3:**
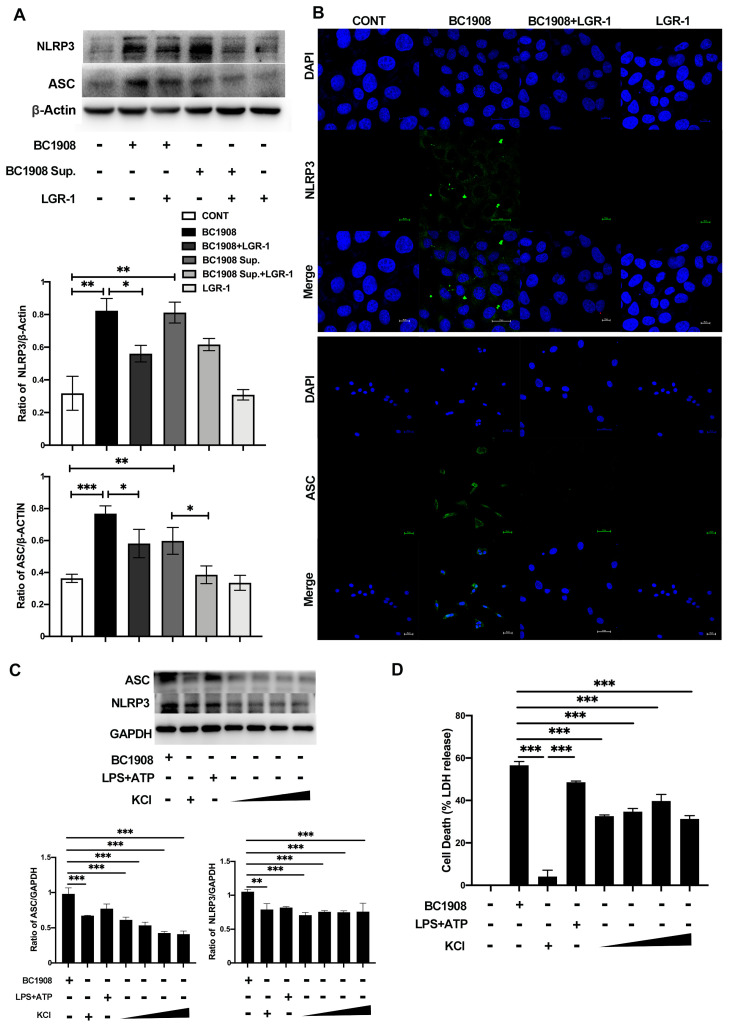
LGR-1 inhibits the activation of cellular inflammatory proteins (NLRP3 and ASC) caused by BC1908 and the supernatant of BC1908. (**A**) The WB results show that BC1908 activates NLRP3 and ASC, and LGR-1 relieves this. (**B**) The results of immunofluorescence show that LGR-1 down-regulated NLRP3 and ASC of cells, caused by BC1908. Scale bar is 25 μm. (**C**) Inhibition of different concentrations of potassium ions on NLRP3 and ASC. (**D**) The exploration of the effect of adding different concentrations of K^+^ to cell LDH. (* *p* < 0.05, ** *p* < 0.01, *** *p* < 0.001).

The inflammatory proteins were quantitatively analyzed by Western blotting and immunofluorescence, and the results are shown in Figure 3A,B. The expression levels of NLRP3 and ASC were significantly increased (*p* < 0.01) in the BC1908 and BC1908 Sup. treatments compared to that of the control cells. Surprisingly, after cells were protected in advance for 3 h with LGR-1 and then exposed to BC1908 and BC1908 Sup., the expression levels of NLRP3 and ASC decreased substantially, especially effective in protecting BC1908-infected cells, which may be related to the protective mechanism of LGR-1. The expression levels of NLRP3 and ASC in the cells supplemented with LGR-1 alone did not change significantly from the control cells. The immunofluorescence results also provided evidence that LGR-1 can relieve the activation of NLRP3 and ASC of cells caused by *B. cereus*.

In Figure 3C, by adding different concentrations of potassium ions in advance, we can observe that the expression levels of NLRP3 and ASC significantly decreased compared to those of the cells infected by BC1908. Moreover, adding 25 mM potassium ions can inhibit the efflux of K^+^ in cells, thereby alleviating the activation of NLRP3 and ASC. In Figure 3D, the results of the LDH indicate that the addition of K^+^ can effectively reduce the cytotoxicity of cells caused by *B. cereus* and decrease intracellular lactate dehydrogenase efflux.

### 3.7. LGR-1 Promotes the Tight Junction of BEECs Infected by B. cereus

**Figure 4:**
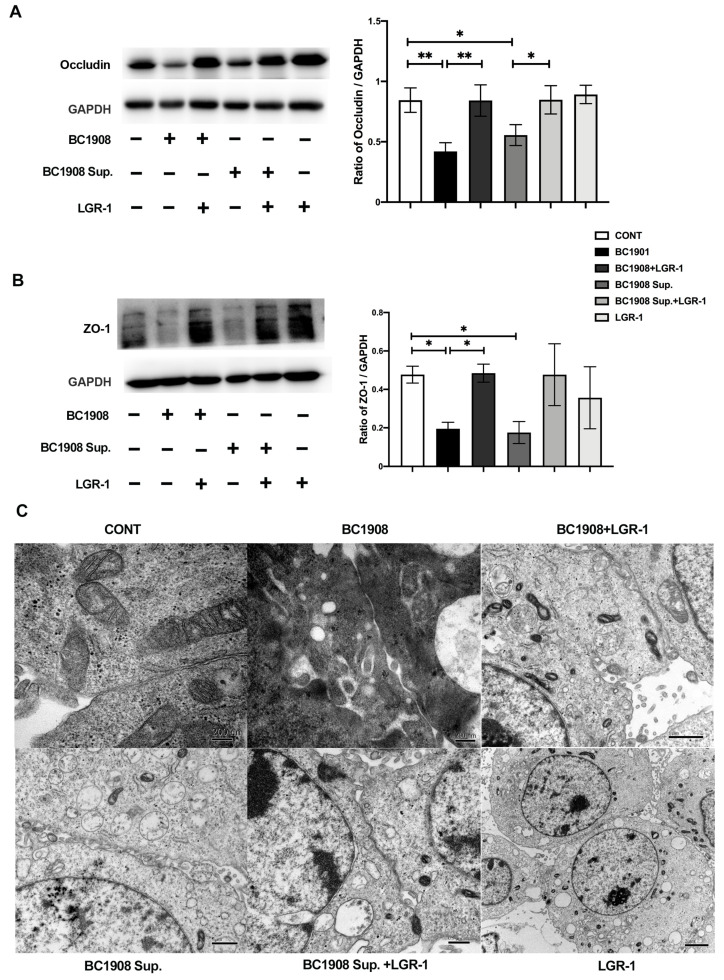
LGR-1 protects the destruction of the tight junction proteins, Occludin (**A**) and ZO-1 (**B**) caused by BC1908 and the supernatant of BC1908. (**C**) Transmission electron microscopy observed the tight junctions between cells with different treatments. (* *p* <0.05, ** *p* < 0.01).

The detection of tight junction proteins, which play an important barrier role to epithelial cells, indicates that both BC1908 and the supernatant of BC1908 have a significantly destructive effect on the tight junction proteins of cells (Figure 4A,B). The expression levels of Occludin and ZO-1 of those cells treated with BC1908 and the supernatant of BC1908 were reduced compared to those in the control cells. More importantly, the expression of the tight junction proteins in cells protected by LGR-1 3 h in advance recovered considerably. Predictably, probiotics had no obvious negative effects on the tight junctions of the cells. In Figure 4C, the scanning electron microscopy results confirm the results of Western blotting, in which LGR-1 alleviated the destruction of the tight junctions of cells caused by BC1908 and BC1908 Sup.

### 3.8. The Pathways of LGR-1 to Protect from Cell Apoptosis Caused by B. cereus

**Figure 5:**
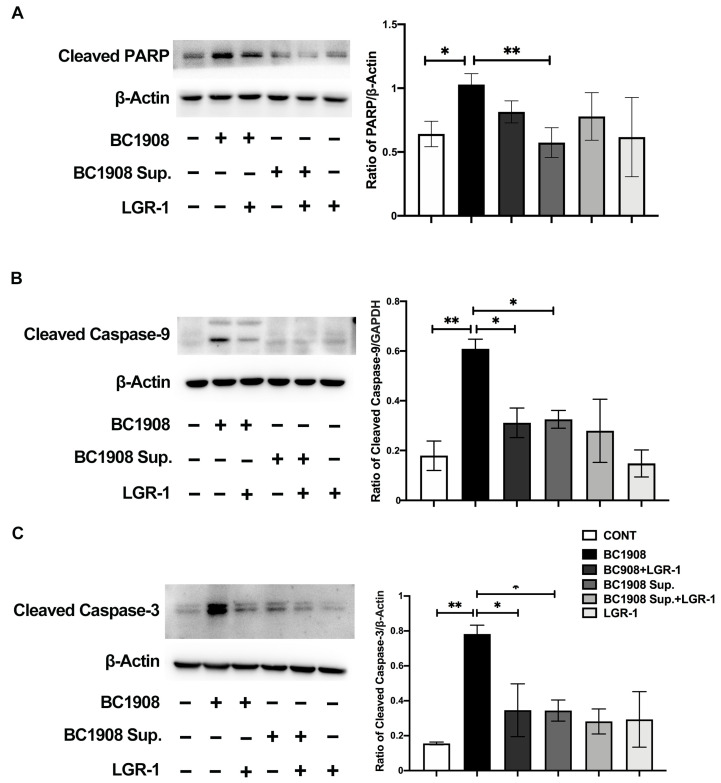
LGR-1 prevents the activation of BC1908 and the BC1908 supernatant on the apoptotic proteins of endometrial epithelial cells. (**A**–**C**) Proof of *B. cereus* activates the apoptosis pathway, and LGR-1 relieves the activation of the apoptosis pathway. (* *p* < 0.05, ** *p* < 0.01).

The expression levels of the apoptosis-related proteins PARP, caspase-9, and caspase-3 were significantly up-regulated in the cells infected by BC1908 and considerably down-regulated in the cells protected by LGR-1 in advance for 3 h (Figure 5A–C). However, the supernatant of BC1908 did not affect the regulation of apoptosis proteins, which resulted in a significant difference in the expression levels of PARP, caspases-9, and caspases-3 between cells infected by BC1908 and treated with the supernatant of BC1908.

**Figure 6:**
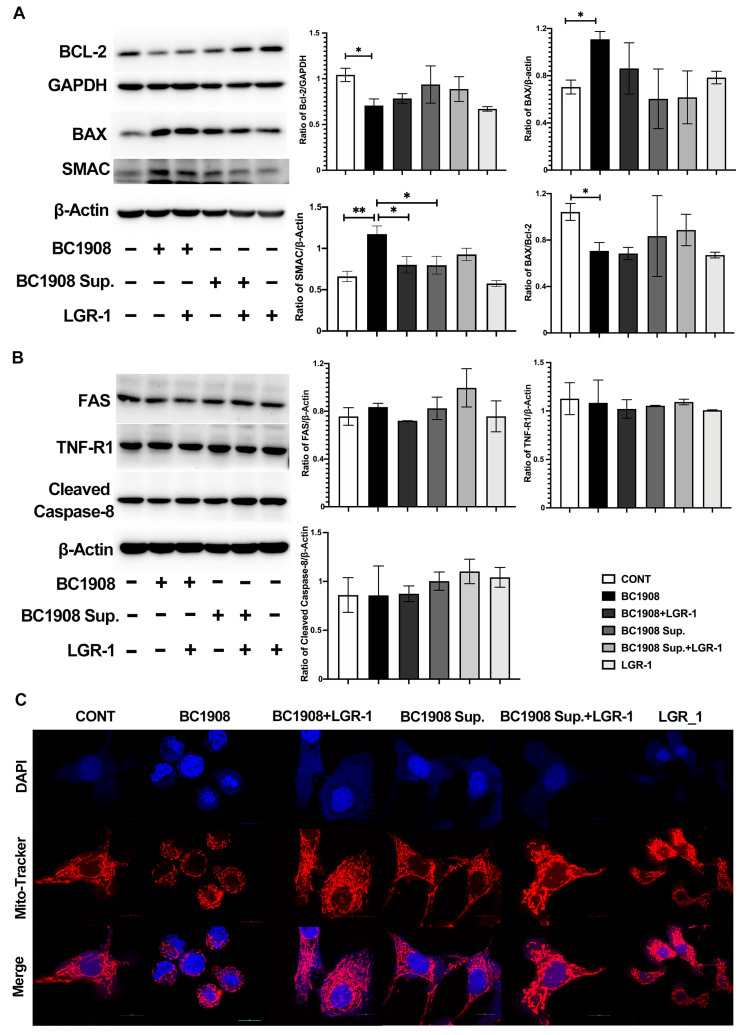
Further study on the apoptosis pathway of endometrial epithelial cells induced by *B. cereus*. (**A**) *B. cereus* activated the apoptotic pathway by the mitochondrial pathway. (**B**) Detection of the exogenous apoptosis pathway indicators, including FAS, TNF-R1, and cleaved caspase-8. (**C**) Immunofluorescence investigated the damage of *B. cereus* on mitochondria and the protective effect of LGR-1 on cell mitochondria. Scale bar is 10 μm. (* *p* <0.05, ** *p* < 0.01).

In order to further explore the pathways of *B. cereus*-activating apoptosis, more apoptosis-related proteins have also been tested (Figure 6). In Figure 6A, by detecting the mitochondrial apoptosis pathway-related pro-apoptotic proteins, which are endogenously apoptosis, the BAX and SMAC expression levels of BC1908-treated cells were significantly up-regulated. Meanwhile, the expression of the apoptosis-inhibiting proteins, BCL-2 and XIAP, were significantly down-regulated. Excitingly, the protection of LGR-1 on cells applied 3 h in advance can effectively alleviate the apoptosis caused by BC1908 on cells. Consistent with the previous results, the supernatants of BC1908 and LGR-1 alone do not affect the regulation of apoptosis-related proteins to epithelial cells. To verify our results, the exogenous apoptosis-related proteins FAS, TNF-R1, and caspase-8 were also tested (Figure 6B).

The expression levels of these proteins did not modulate due to the infection of BC1908 or the supernatant of BC1908. Of course, the addition of LGR-1 did not change the expression of these proteins. The results of immunofluorescence (Figure 6C) further confirmed that BC1908 damages cell mitochondria and then activates caspase-9 and caspase-3, causing cell apoptosis. The nucleus of cells infected by BC1908 also shrank and stained unevenly, indicating the appearance of the nucleus. The condition of the nuclei of cells protected by LGR-1 in advance for 3 h improved, and the morphology of the mitochondria was also filamentous or reticulated. Similarly, the supernatant of BC1908 had a practically very weak destructive effect on the mitochondria and the nucleus.

## 4. Discussion

The history of human development is also the history of the human struggle against bacterial infections. The morbidity and fatality rates caused by bacterial infection accompanied mankind and livestock history until the emergence of antibiotics. Antibiotic therapies are still the primary treatment to combat bacterial infections, which are usually long-lasting and difficult to remove completely due to biofilm production and other protection [1]. The overuse of antibiotics has led to the emergence of drug resistance and other strategies to survive in adverse conditions, which has emerged as antibiotic treatment and the host autoimmunity failing to eliminate drug-resistant bacteria, resulting in the recurrence of and susceptibility to infection and slow restoration. Those bacteria that survived from drug treatment and purging of the host can utilize the intrauterine environment for benefits. For example, the warm and humid environment of the endometrium can provide a suitable environment for bacteria, and the host can be easily damaged again. Previous reports have also proved that several extracapsular bacteria can invade and reproduce in epithelial cells under specific conditions [25,26]. These infections are often chronic and are never fully cleared by antibiotic treatment because bacteria can persist in biofilms or other protected niches [27]. The persistent infestation of cells by these recalcitrance bacteria is the cause of the failure of antibiotic treatment [28,29,30,31]. Alternatives to compete against bacterial infections are urgently needed.

The vaginal microbiota, which is dominated by *Lactobacilli*, plays an important role in maintaining female health [32,33]. Bacterial infections in the uterus and vagina postpartum can cause microbial disorders and affect the involution, resulting in the disease of dairy cows. Probiotics, as a substitute for antibiotics, have been widely used to combat diseases caused by bacterial infections. *Lactobacilli* are safe probiotics that antagonize pathogens, have anti-inflammatory (anticolitic and antivaginitic) effects [34,35,36,37], and regulate immunity in vivo and in vitro. Probiotics may be related to the regulation of the immune response in the uterus and vagina, rather than directly competing or killing pathogenic microorganisms [37]. However, the existence of probiotics, including the bacteriocin secreted by probiotics, may have a certain inhibitory effect on pathogenic bacteria, which is consistent with previous reports. Pathogens can be restricted by *Lactobacillus* through a series of strategies, including immune system activation and the secretion of specific antibacterial proteins [38,39]. It has been indicated that *Lactobacillus* species can exert an anti-inflammatory effect by regulating the level of cytokines involved in the inflammatory response.

Pore-forming toxins as weapons secreted from pathogens can break up the epithelial barrier by inducing apoptosis in epithelial cells [40,41,42,43]. Our results revealed that LGR-1 could effectively alleviate the mitochondrial-modulated apoptosis of primary epithelial cells caused by *B. cereus*. The strain used in this experiment has no regulatory effect on the exogenous apoptosis pathway. The permeabilization of mitochondrial membranes releases several mitochondrial proteins, such as SMAC [44]. By damaging the mitochondria, BC1908 increases the permeability of the mitochondrial membrane, causing the efflux of BAX and SMAC from the mitochondria, activating caspase-9 and then caspase-3. BAX actively induces cytochrome *c* release from the mitochondria within cells and in cell-free systems, both of which are inhibited by Bcl-2 family members [45]. The latter triggers a caspase cascade, which begins with the initiator caspase, caspase-9, and culminates in activating the effectors, caspase-3 and caspase-7 [46]. Remarkably, those processes correspond to a BAX-mediated reduction of the inhibitors of apoptosis (IAP) family members cIAP1 and XIAP (X-linked IAP) [47]. SMAC is believed to function as a neutralizer of caspase inhibitors, and mass spectrometry analysis has identified XIAP as a predominant SMAC-binding protein [48]. A key feature of regulated apoptosis is the release of SMAC from mitochondria and the subsequent release of caspases from inhibition [49]. Our results also indicate that the protective effect of LGR-1 against apoptosis is regulated by the mitochondrial pathway.

In this study, *B. cereus* caused initial damage by K^+^ efflux, leading to the activation of the NLRP3 inflammatory pathway. After activation, these sensors recruit the adaptor protein ASC to form a functional inflammasome complex [50,51,52]. Different from Gram-negative bacteria, Gram-positive bacteria mainly affect cell function, metabolism, and physiological functions through toxins secreted by virulence genes to benefit bacterial replication and transmission [53]. Some virulence genes of *B. cereus* were detected in our research, including NHE, HBL, *cytK,* and *inhA*, which can form pores on mammalian cell membranes [54]. Previous studies have revealed that HBL- and NHE-induced activation of the NLRP3 inflammasome requires K^+^ efflux [50]. LGR-1 effectively alleviated the inflammatory response caused by *B. cereus,* of which this alleviation may also come from the control of K^+^ efflux. Previous research has demonstrated that preventing and treating the pertinence of toxin-secreting bacterial infections may be an efficient and general approach.

When associated with an inflammasome-priming stimulus to promote precursor IL-1β expression and raise NLRP3 levels, the activation of BAX triggers caspase-1 and IL-1β processing and secretion [47]. Another report showed that the membrane damage or K^+^ channel activation is prompted by the BAX signaling pathway via caspase-3 and -7, leading to NLRP3 inflammasome formation [47]. This view also coincides with the characteristics of the model building in this experiment. The damage of cells begins when the toxins secreted by *B. cereus* perforate the cell membrane to excrete K^+^, which leads to activation of the BAX and the NLRP3 signaling pathways. However, why K^+^ efflux can activate the NLRP3 pathway is still an unsolved mystery. The addition of K^+^ reduces the efflux of lactate dehydrogenase in cells, which may also be a reason for inhibiting the activation of NLRP3 and BAX. Moreover, incubating LGR-1 in advance can also effectively limit the activation of the cellular NLRP3 and BAX signal pathways caused by *B. cereus*, which may be because LGR-1 can effectively prevent K^+^ efflux in cells. The ability of LGR-1 to limit the HBL and NHE toxins, which could be inserted into the mammalian cell membrane to form pores, thereby mediating cellular leakage and lysis, may need further investigation.

## 5. Conclusions

Herein, we reported that if the emergence of multidrug-resistant *B. cereus* cannot be effectively and safely prevented and treated, the threat to food safety and even human health will pose a serious challenge to animal husbandry. Probiotics, an alternative to antibiotics, such as *L. rhamnosus* GR-1, can improve cell viability via the characteristics of immunomodulation, apoptosis inhibition, and maintenance of the integrity of endometrial epithelial cells. Moreover, this study also provides a theoretical basis for future practical applications of *L. rhamnosus* GR-1 to prevent and treat bovine endometritis.

## Figures and Tables

**Table 1:** The sequencing of virulence gene primers.

Gene Name		Primer Sequences (5′–3′)	Fragment Size (bp)
NHEA	F	CGGGATCCACGAGTTGCTTCATTCCTGTAAGC	1132
	R	CCCTCGAGTTAATGTACTTCAACGTTTGTAACGTAATCTTCAAAT	
NHEB	F	CGGAATTCAATATTATGCCGGCTCATACGTATGCA	1162
	R	CCCTCGAGTTATGCTTTTTTCGTATCTACTACTTTAATATCTTC	
NHEC	F	CGGAATTCATGCCGGCTCATACGTAT	1156
	R	CCCTCGAGTTATGCTTTTTTCGTATCTACTACTTTAATATCTTCA	
HBLA	F	CGGGATCCGCAGTCATACCAATAGAAACTTTTGC	1351
	R	CCCTCGAGTCAGTTCATTATATTTTGTACTTTGTCTTTATACAC	
HBLB	F	CGGAATTCTCACCAGTAACAACTTTTGCAAGTGAA	1072
	R	CCCTCGAGCTATTTTTGTGGAGTAACAGTTTCCACTTTT	
HBLD	F	CGGGATCCGCATTTGCACAAGAAACGACCG	1156
	R	CCCTCGAGCTACTCCTGTTTAAAAGCAATATCTTTTGAAATGAA	
*Hly* *III*	F	CGGAATTCGCAATTACACATGGTATCGGTG	619
	R	CCCTCGAGTTATGCTGTAGGTAAGACATAAAAGAGTACA	
*inhA*	F	CGGAATTCATGAGTGCTCCGTTAGCATATGCA	2344
	R	CCCTCGAGTTAACGTTTAATCCAAACAGCGCCTGC	
*hlyA*	F	CGGAATTCGCCATTATGGCCGGACT	778
	R	CCCTCGAGTTATTCCCCTTTCCCTTTTTGTTTTAG	
*cytk*	F	CGGAATTCCCTGCTACTTACGCTCAAAC	949
	R	CCCTCGAGTTATTTTTTCTCTACTAATTTCTTATTCTTCCAATCTAG	

**Table 2:** Antimicrobial susceptibility profile of BC1908.

Strain	Antibiotics										
	CZ	KAN	AMP	STR	AZM	AMC	ENR	TET	GM	CIP	MEM
BC1908	R	R	R	S	R	R	S	R	R	R	R

CZ, cefazolin; KAN, kanamycin; AMP, ampicillin; STR, streptomycin; AZM, azithromycin; AMC, amoxicillin; ENR, enrofloxacin; TET, tetracycline; GM, gentamicin; CIP, ciprofloxacin; MEM, meropenem; S, susceptible; I, intermediate; R, resistant.

## Supplementary Materials

The following supporting information can be downloaded at: https://www.mdpi.com/article/10.3390/microorganisms10010137/s1, Figure S1: Explore the cytotoxicity of *B. cereus* and the protective effect of LGR-1 on cells. Figure S2: To explore the virulence gene carrier rate of clinical isolates of *Bacillus cereus*.
