# Supplementary material for: Lactobacillus rhamnosus Ameliorates Multi-Drug-Resistant Bacillus cereus-Induced Cell Damage through Inhibition of NLRP3 Inflammasomes and Apoptosis in Bovine Endometritis
Source: Microorganisms. 2022 Jan 10;10(1):137. doi: 10.3390/microorganisms10010137 (PMC8777719; doi:10.3390/microorganisms10010137)
Supplement: Supplementary file 1 [file microorganisms-10-00137-s001.zip › microorganisms-1544414-supplementary.pdf]

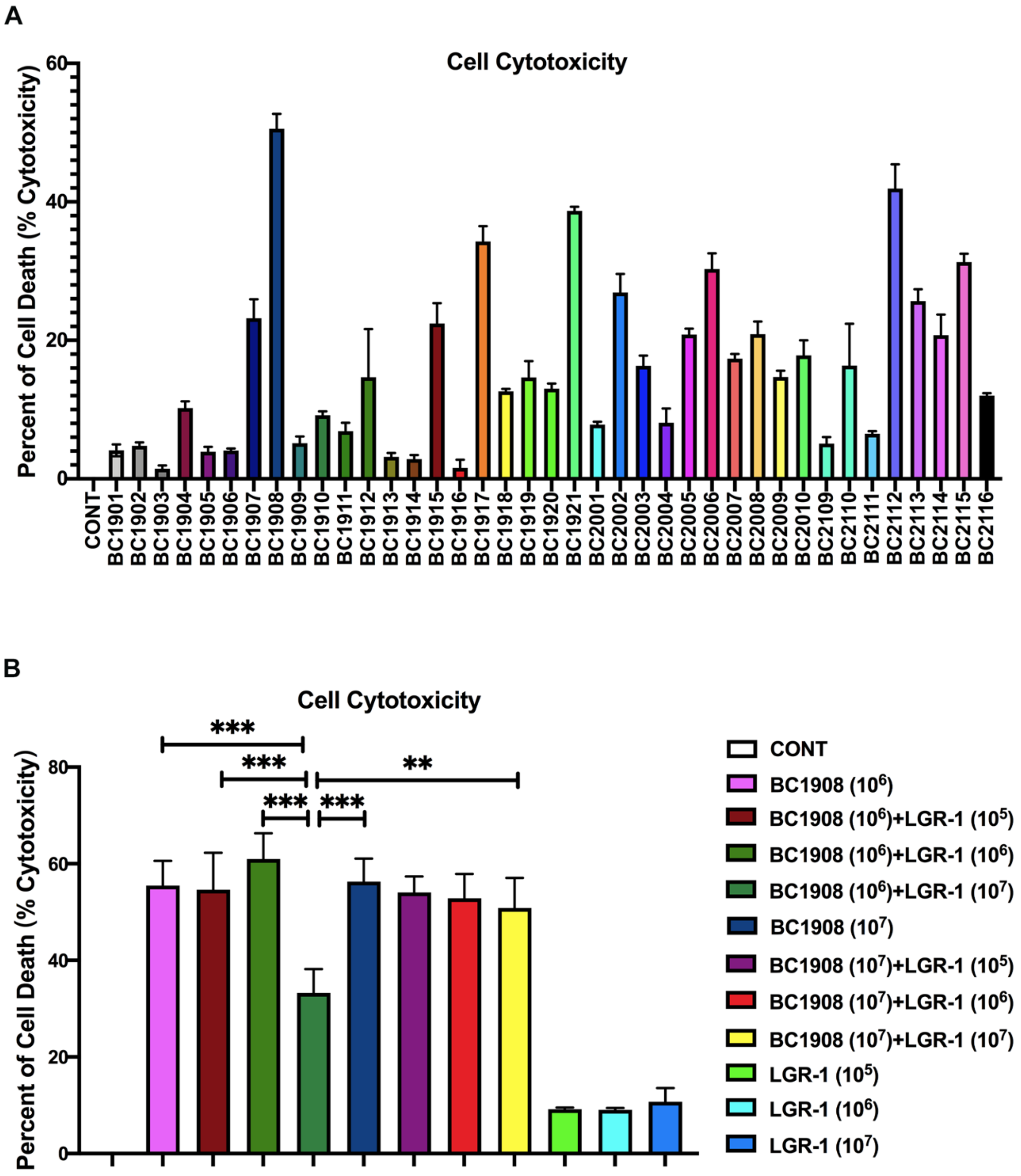

Supplementary Figure S1. Explore the cytotoxicity of *B. cereus* and the protective effect of LGR-1 on cells. (A) Toxic effects of *B. cereus* on endometrial epithelial cells after three hours of action. (B) Using different BC1908 concentrations and different LGR-1 concentrations to explore the least protective effect of LGR-1 on *B. cereus*.

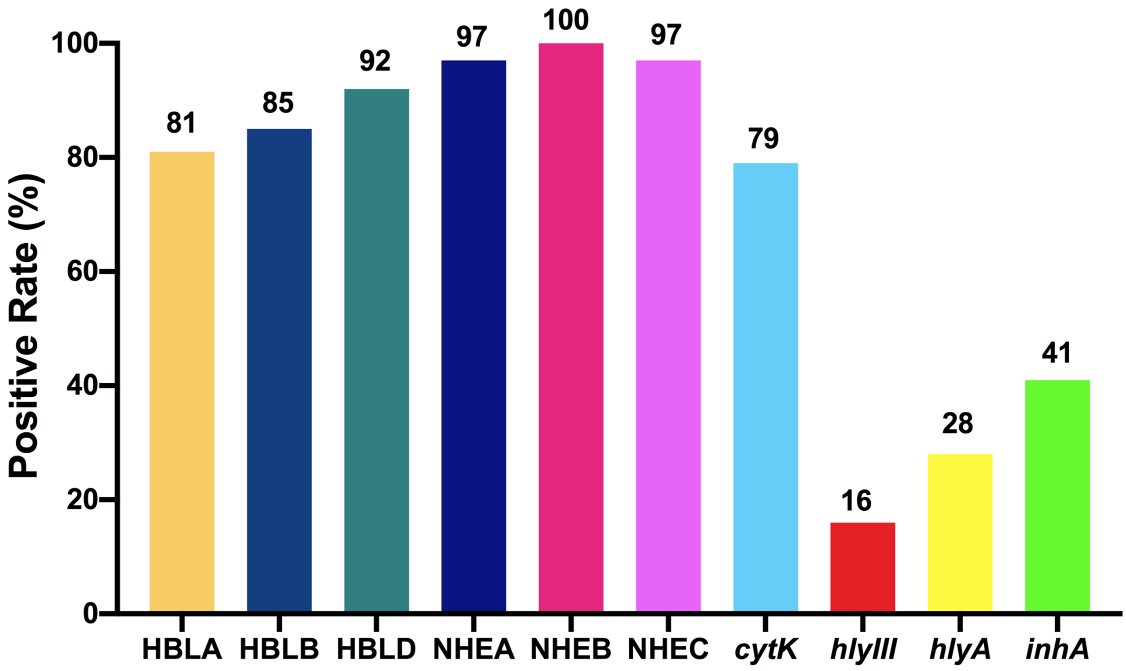

Supplementary Figure S2. To explore the virulence gene carrier rate of clinical isolates of *Bacillus cereus*. The numbers above the bar graph represent the positive rates of virulence genes in all clinical isolates of *B. cereus*.
